# Modeling and Analysis of Torsional Stiffness in Rehabilitation Robot Joints Using Fractal Theory

**DOI:** 10.3390/ma18122866

**Published:** 2025-06-17

**Authors:** Shuaidong Zou, Wenjie Yan, Guanghui Xie, Renqiang Yang, Huachao Xu, Fanwei Sun

**Affiliations:** 1 College of Smart Health, Chongqing Polytechnic University of Electronic Technology, Chongqing 401331, China; 2 State Key Laboratory of Mechanical Transmission for Advanced Equipment, Chongqing University, Chongqing 400044, China; 3 School of Engineering, University of Auckland, Auckland 0632, New Zealand

**Keywords:** rehabilitation robot joint, torsional stiffness, fractal theory, planetary traction drive

## Abstract

The torsional stiffness of rehabilitation robot joints is a critical performance determinant, significantly affecting motion accuracy, stability, and user comfort. This paper introduces an innovative traction drive mechanism that transmits torque through friction forces, overcoming mechanical impact issues of traditional gear transmissions, though accurately modeling surface roughness effects remains challenging. Based on fractal theory, this study presents a comprehensive torsional stiffness analysis for advanced traction drive joints. Surface topography is characterized using the Weierstrass–Mandelbrot function, and a contact mechanics model accounting for elastic–plastic deformation of micro-asperities is developed to derive the tangential stiffness of individual contact pairs. Static force analysis determines load distribution, and overall joint torsional stiffness is calculated through the integration of individual contact contributions. Parametric analyses reveal that contact stiffness increases with normal load, contact length, and radius, while decreasing with the tangential load and roughness parameter. Stiffness exhibits a non-monotonic relationship with fractal dimension, reaching a maximum at intermediate values. Overall system stiffness demonstrates similar parameter dependencies, with a slight decrease under increasing output load when sufficient preload is applied. This fractal-based model enables more accurate stiffness prediction and offers valuable theoretical guidance for design optimization and performance improvement in rehabilitation robot joints.

## 1. Introduction

**Figure 1:**
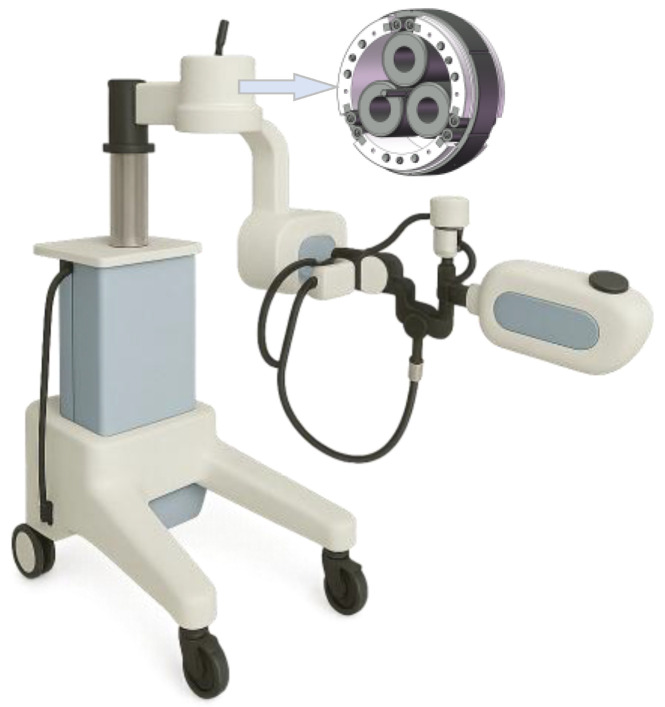
Rehabilitation robot and its joint transmission system.

With ongoing advancements in the interdisciplinary integration of biomedical and mechanical engineering, The rehabilitation robot shown in Figure 1 have become increasingly prevalent in clinical practice [1,2]. Recent developments in sensor technology, control algorithms, and human–machine interfaces have further accelerated this trend [3,4,5], with particular progress in upper limb rehabilitation systems [6] and lower limb exoskeletons [7]. These devices deliver precisely controlled assistive forces to support patients with motor impairments (particularly in the upper and lower limbs, including the shoulders, wrists, and fingers) resulting from stroke, neurological disorders, or injuries. They enable patients to perform repetitive, quantitative rehabilitation training, promoting functional recovery and facilitating neural tissue repair and regeneration. Ensuring safe and effective physical human–robot interactions is essential in the design of rehabilitation robots [1], as joint performance directly affects motion accuracy, stability, and user comfort [8]. Traction drives (TDs), which transmit power through frictional forces between smooth rolling elements (typically under lubrication), offer significant potential in rehabilitation robotics, where precision motion and high-quality human–machine interaction are essential. Their inherent advantages, including smooth operation, low noise, and zero backlash, make them especially suitable for such applications [9,10]. Among various configurations, the planetary traction drive (PTD) is notable for its compact design and ability to achieve high transmission ratios [11].

Torsional stiffness is a fundamental parameter that defines the performance of PTD systems. It directly affects the transmission system’s positioning accuracy [12] and dynamic response characteristics, such as natural frequency and stability [13]. Moreover, it plays a critical role in a rehabilitation robot’s ability to replicate natural human movements, deliver high-fidelity force feedback, and maintain stability during user interaction [14]. Accurate prediction and understanding of joint stiffness are essential for the design of both fixed-stiffness joints and variable-stiffness actuators, which require active compliance adjustment [15]. However, accurately predicting the torsional stiffness of PTDs remains a challenge. Traditional analysis methods often rely on Hertzian contact theory [16], which assumes ideally smooth contact surfaces. This assumption deviates significantly from the reality of engineering surfaces, which typically exhibit roughness at the microscopic scale. Surface roughness results in real contact occurring only at the peaks of a few micro-asperities [17], making the real contact area much smaller than the nominal contact area. This discrepancy is particularly evident under low-to-medium loads, where contact pressure and stiffness behavior significantly differ from Hertzian predictions [18,19]. Although statistical models such as the Greenwood–Williamson model account for surface height randomness, their core assumption of “independent asperity action” breaks down at high contact densities [20]. Furthermore, these models typically rely on conventional roughness parameters such as $R_{a}$ and $R_{q}$, which are scale-dependent and fail to fully represent the multi-scale surface features that critically influence contact performance [21]. Consequently, stiffness predictions based on traditional models often fall short of meeting the stringent design requirements of high-performance rehabilitation robots.

In recent years, fractal geometry theory, pioneered by Mandelbrot [22], has emerged as a powerful mathematical framework for describing the complex, irregular, self-similar, or self-affine surface topographies commonly found in both nature and engineering. The Weierstrass–Mandelbrot (W–M) function [23,24], in particular, is widely used to generate and characterize surface profiles with multi-scale features. This approach has been successfully applied in various tribological studies [25,26] and contact mechanics analyses [27,28]. Unlike traditional roughness parameters, the fractal dimension D and fractal roughness parameter G [24,29] are scale-independent descriptors that quantify surface complexity (frequency content) and amplitude, respectively, offering a more comprehensive basis for surface characterization. Fractal contact mechanics models, especially asperity-based approaches such as the Majumdar-Bhushan model [30] and its successors, have been developed. These models integrate fractal surface descriptions with analyses of elastic, elastic–plastic, and plastic deformation of individual micro-asperities under contact loads [31]. By summing the contributions of all contacting asperities, the models enable the calculation of macroscopic contact forces, real contact areas, and both normal and tangential contact stiffness.

Despite significant advancements in fractal contact mechanics and extensive research on the design, performance, and load distribution of PTD systems [11,32,33], the systematic application of advanced fractal contact models to accurately predict the overall torsional stiffness of PTD systems remains limited. Existing analyses often rely on simplified contact models (such as Hertzian theory) or experimental measurements [12], which do not fully exploit the potential of fractal theory to account for the effects of surface roughness. Recent studies have shown promising results in applying advanced contact models to gear systems [34] and bearing assemblies [35], but systematic application to PTD torsional stiffness remains limited. This highlights a clear gap in current research: the absence of an integrated modeling framework that combines an advanced fractal contact stiffness model—capable of capturing surface roughness, elastic–plastic deformation, and tangential effects—with a comprehensive PTD system-level model. Such a model should incorporate multiple contact pairs (e.g., sun-planet, planet-ring), component flexibility (e.g., shafts, bearings, planet carrier), and load distribution characteristics to enable accurate prediction of overall torsional stiffness.

This paper aims to bridge this gap by proposing a fractal theory-based methodology for modeling and analyzing the overall torsional stiffness of a PTD system used in rehabilitation robots. The approach begins with a detailed static force analysis of the PTD system to determine the internal load distribution. Next, the W–M function is employed to characterize the surface topography of key contact interfaces, and a fractal contact mechanics model that accounts for the elastic–plastic deformation of micro-asperities is developed to calculate the normal and tangential stiffness for each contact pair. Finally, based on the system’s structural topology, the stiffnesses of various components are integrated using series–parallel combination rules (considering speed ratio conversion [12,36]) to obtain the overall torsional stiffness of the system. Afterward, parametric simulation analyses are conducted to thoroughly investigate the effects of factors such as normal and tangential loads, fractal parameters (D, G), geometric dimensions (R, L), material properties (E, H), and the friction coefficient (μ) on both contact stiffness and overall system torsional stiffness. This research aims to provide a more accurate and realistic tool for predicting PTD joint stiffness, offering valuable theoretical insights and engineering guidance for the design optimization, performance prediction, and control strategy development of high-performance rehabilitation robots.

## 2. Force Analysis of the Joint Traction Drive System

**Figure 2:**
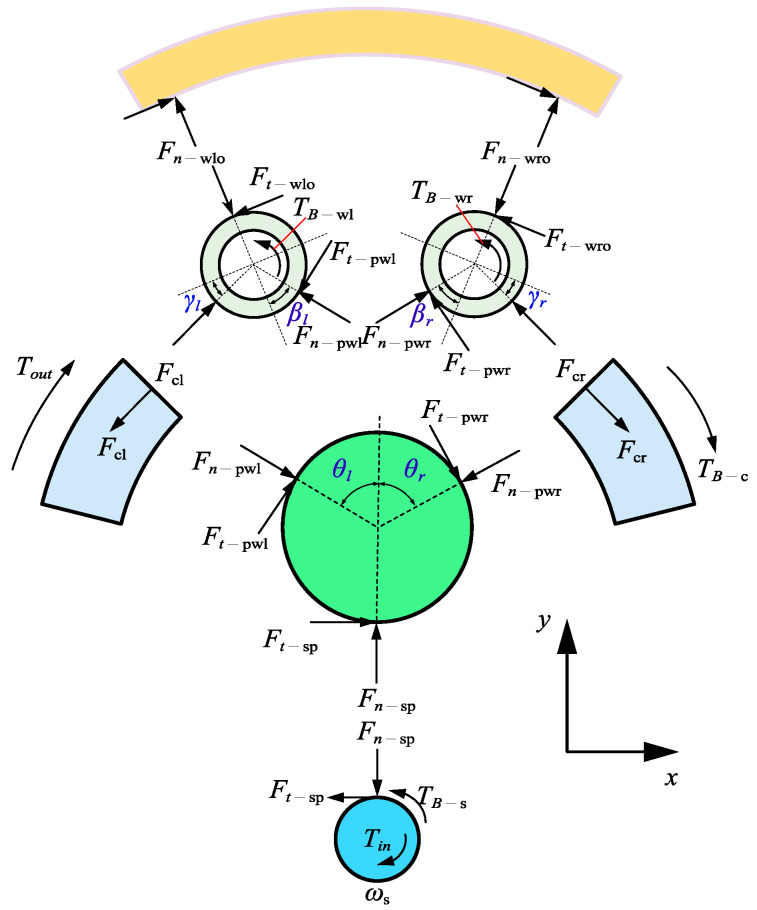
Schematic of forces.

When the rehabilitation robot joint operates stably, contact forces are generated between the sun roller and the three planet rollers within the TD system. These contact forces can be decomposed into normal and tangential components. The resultant vector and the resultant moment of the three normal forces are both zero, as shown in Figure 2. When an input torque $T_{in}$ is applied, the tangential forces counterbalance the bearing friction torques and $T_{in}$. Taking the centre of the sun roller as the origin, with the horizontal direction as the *x*-axis and the vertical direction as the *y*-axis, the moment equilibrium equation for the sun roller is given as follows:

(1) $$T_{in}-2T_{B-s}-3F_{t-sp}\cdot R_{s}=0$$

where $T_{B}$ represents the bearing friction torque; $F_{n}$ and $F_{t}$ denote the normal and tangential forces, respectively; and R represents the roller radius. The subscripts s, p, wl, wr, cl, cr, and o refer to the sun roller, planet roller, left loading roller, right loading roller, left planet carrier, right planet carrier, and outer ring, respectively. The subscript sp indicates the interaction between s and p, and a similar notation applies to other pairs.

The planet roller experiences contact forces from the sun roller and the left and right loading rollers. These forces are counterbalanced by the planet roller’s inertia force. The force and moment equilibrium equations for the planet roller are as follows:

(2) $$\left\{\begin{array}{c} F_{t-sp}+F_{t-pwr}\cos\theta_{r}-F_{n-pwr}\sin\theta_{r}+F_{n-pwl}\cos\theta_{l}+F_{t-pwl}\cos\theta_{l}=0\\ F_{n-sp}-F_{t-pwr}\sin\theta_{r}-F_{n-pwr}\cos\theta_{r}-F_{n-pwl}\cos\theta_{l}+F_{t-pwl}\sin\theta_{l}=m_{p}{\omega_{c}}^{2}\left(R_{s}+R_{p}\right)\\ \left(F_{t-pwr}+F_{t-pwl}-F_{t-sp}\right)R_{p}=0 \end{array}\right.$$

where $\theta_{r}$ and $\theta_{l}$ are the angles formed between the normal load vectors from the right and left loading rollers to the planet roller and the *y*-axis, respectively; m represents the mass of the roller; and $\omega_{c}$ is the angular velocity of the planet carrier.

The force and moment equilibrium equations for the left and right loading rollers are as follows:

For the left loading roller:

(3) $$\left\{\begin{array}{c} F_{cl}\cos\gamma_{l}-F_{n-pwl}\sin\beta_{l}-F_{t-pwl}\cos\beta_{l}-F_{t-wlo}=0\\ F_{cl}\sin\gamma_{l}+F_{n-pwl}\cos\beta_{l}-F_{t-pwl}\sin\beta_{l}-F_{n-wlo}=m_{wl}{\omega_{c}}^{2}(R_{o}-R_{wl})\\ (F_{t-wlo}-F_{t-pwl})\cdot R_{wl}+2T_{B-wl}=0 \end{array}\right.$$

For the right loading roller:

(4) $$\left\{\begin{array}{c} F_{cr}\cos\gamma_{r}-F_{n-pwr}\sin\beta_{r}-F_{t-pwr}\cos\beta_{r}+F_{t-wro}=0\\ F_{cr}\sin\gamma_{r}+F_{n-pwr}\cos\beta_{r}-F_{t-pwr}\sin\beta_{r}-F_{n-wro}=m_{wr}{\omega_{c}}^{2}(R_{o}-R_{wr})\\ (F_{t-wro}-F_{t-pwr})\cdot R_{wr}+2T_{B-wr}=0 \end{array}\right.$$

where $\beta_{l}$, $\beta_{r}$, $\gamma_{l}$, and $\gamma_{r}$ are the load application angles.

The moment equilibrium equation for the planet carrier is as follows:

(5) $$3(F_{cl}\cos\gamma_{l}-F_{cr}\cos\gamma_{r})(R_{o}-R_{wr})-2T_{B-c}-T_{out}=0$$

## 3. Modeling of Tangential Stiffness of Contact Pairs Based on Fractal Theory

The accurate description of surface topography is crucial for understanding contact mechanics, friction, and lubrication phenomena, all of which stem from interactions at the micro-asperity level. While statistical parameters such as $R_{a}$ and $R_{q}$ are commonly used, they are significantly affected by scale dependency, which varies with measurement conditions.

Hertzian theory assumes perfectly smooth surfaces and predicts contact behavior based on macroscopic geometry alone [16]. However, as extensively documented in the literature [18,19], real engineering surfaces exhibit significant roughness that substantially affects contact stiffness. Classical statistical approaches like the Greenwood-Williamson model use scale-dependent roughness parameters ($R_{a}$, $R_{q}$) that vary with measurement conditions [21]. As highlighted by Majumdar and Bhushan [30], these limitations become pronounced when characterizing the multi-scale nature of real surfaces. The fractal parameters D and G used in our model are scale-independent, providing more consistent surface characterization. Unlike conventional approaches that rely on single-scale characterization, fractal geometry captures the self-similar nature of engineering surfaces across multiple scales [22], which is particularly relevant for precision applications like rehabilitation robots where accurate stiffness prediction is crucial for performance and safety.

Fractal geometry offers a scale-independent alternative that captures the self-similar or self-affine nature of many real surfaces. It utilizes the fractal dimension D (representing complexity) and the fractal roughness G (representing amplitude) to provide a more consistent surface representation across different measurement scales, thus overcoming the limitations of traditional statistical methods.

The W–M function, which generates fractal curves that are continuous everywhere but not differentiable anywhere and display self-affine characteristics, is commonly used to model contact surface topography. The mathematical expression for a two-dimensional surface profile described by the W–M fractal function is provided in [37], as follows:

(6) $$z(x)=L(\frac{G}{L}{)}^{D-1}(\ln\gamma {)}^{1/2}\sum_{n=n_{l}}^{n=n_{h}}\gamma^{(D-2)n}\left[\cos\phi_{n}-\cos(\frac{2\pi \gamma^{n}x}{L}-\phi_{n})\right],\;1<D<2$$

The contact between two rough surfaces can be equivalently modelled as the contact between an equivalent rough surface and an ideally rigid flat. To describe the state of the rough surface after deformation, its pre-deformation morphology must first be characterized. Given that any theoretical model is based on specific assumptions, the analysis in this paper relies on the following hypotheses:

1. The contact between two rough surfaces is simplified to that between an equivalent rough surface and an ideally rigid flat;
2. The surface roughness is statistically isotropic;
3. All micro-asperities share a common base plane, which is assumed to be fixed and does not shift with the applied normal load. As the normal load increases, the contact plane moves toward the base plane;
4. Work hardening and interactions between deforming micro-asperities are neglected during the deformation process.

Under the assumption that a micro-asperity can be modelled as a hemisphere with a radius of curvature R, the profile equation of a single micro-asperity before deformation is given by

(7) $$z_{0}(x)=G^{D-1}{\left(\ln\gamma \right)}^{\frac{1}{2}}l^{2-D}\cos(\frac{\pi x}{l}),\;-\frac{l}{2}<x<\frac{l}{2}$$

where l is the base length of the micro-asperity; D is the fractal dimension; G is the fractal roughness parameter; and γ is a scaling parameter, typically taken as 1.5 for isotropic surfaces.

Since the base length l = 2r, where r is the radius of the micro-asperity base after deformation, the previous equation can be rewritten as follows:

(8) $$z_{0}(x)=G^{D-1}{\left(\ln\gamma \right)}^{\frac{1}{2}}{\left(2r\right)}^{2-D}\cos(\frac{\pi x}{2r}),\;-r<x<r$$

The radius of curvature R at the summit of the micro-asperity before deformation can be expressed as follows:

(9) $$R=\left|1/{\left(\frac{d^{2}z_{0}(x)}{{dx}^{2}}\right)}_{x=0}\right|={\left(\sqrt{\ln\gamma }\pi^{2}G^{D-1}\right)}^{-1}{\left(2r\right)}^{D}$$

According to fractal theory, the distribution of micro-asperity contact areas on a fractal surface follows a pattern similar to the area distribution of islands in an archipelago. The number of micro-asperities, n(a), with a contact area a on a planar junction is related to the fractal parameters as follows:

(10) $$n\left(a\right)=\frac{D}{2}\psi^{(2-D)/2}a_{l}^{D/2}a^{-(D+2)/2}$$

where ψ is the domain extension factor [26], satisfying $\psi^{(2-D)/2}-{(1+\psi^{-\frac{D}{2)}}}^{(D-2)/D}=(2-D)/D$; a is the contact area of a micro-asperity; and $a_{l}$ is the maximum contact area of a micro-asperity.

For contact between two cylindrical surfaces, the geometry of the surfaces and the magnitude of the normal load influence both the contact area and the distribution of micro-asperities. This contact between cylindrical surfaces results in fewer contacting micro-asperities than the contact between two flat surfaces under similar conditions [38]. Therefore, a contact factor λ is introduced to account for this effect [39], as follows:

(11) $$\lambda ={\left[\frac{{\left(\frac{4B}{\pi E}\frac{R_{1}R_{2}}{R_{1}\pm R_{2}}\right)}^{\frac{1}{2}}}{\pi \left(R_{1}\pm R_{2}\right)}\right]}^{\left(\frac{1}{R_{1}}\pm \frac{1}{R_{2}}\right)}$$

Consequently, the size distribution function n(a) for the contact between two cylindrical surfaces can be expressed as follows:

(12) $$n(a)=\lambda \frac{D}{2}\psi^{(2-D)/2}a_{l}^{D/2}a^{-(D+2)/2}$$

where λ is the contact factor; $R_{1}$ and $R_{2}$ are the radii of the two cylinders; B is the contact length; and E is the equivalent elastic modulus, defined as $1/E=(1-{v_{1}}^{2})/E_{1}+(1-{v_{2}}^{2})/E_{2}$, where $v_{1}$, $v_{2}$, and $E_{1}$, $E_{2}$ are the Poisson’s ratios and Young’s moduli of the contacting materials, respectively.

During the elastic deformation stage, for a micro-asperity with radius of curvature R contacting a smooth rigid flat, the contact area ae and the normal load $F_{e}$ corresponding to a deformation ω are given as follows:

(13) $$a_{e}=\pi R\omega$$

(14) $$F_{e}=\frac{4}{3}ER^{1/2}\omega^{3/2}$$

When a micro-asperity reaches the fully plastic deformation stage, the normal load $F_{p}$ is linearly related to the contact area $a_{p}$, as follows:

(15) $$a_{p}=2\pi R\omega$$

(16) $$F_{p}=Ha_{p}$$

where $a_{e}$ and $a_{p}$ are the micro-asperity contact areas in the elastic and fully plastic deformation stages, respectively; $F_{e}$ and $F_{p}$ are the corresponding normal loads; ω is the micro-asperity deformation; K is the material hardness coefficient; H is the material hardness; and R is the micro-asperity radius of curvature.

Substituting the expressions for R and ω (derived from previous equations) yields the relationship between the load and contact area for a single micro-asperity during the elastic deformation stage:

(17) $$F_{e}=\frac{4\sqrt{\pi }}{3\left(3-2D\right)}\times 2^{-D}E\pi^{\frac{D+1}{2}}\sqrt{\ln\gamma }G^{D-1}a^{\frac{3-D}{2}}$$

From the plastic deformation equation, the relationship between the load and contact area for a single micro-asperity during the plastic deformation stage is given as follows:

(18) $$F_{p}=Ha$$

The critical contact area $a_{c}$, marking the transition from elastic to elastic–plastic deformation, is given as follows [40]:

(19) $$a_{c}=G^{2}{\left(\frac{2E}{H}\right)}^{\frac{2}{D-1}}$$

The total normal load $F_{n}$ generated by micro-asperities in various deformation stages at the contact interface is calculated by integrating the product of the normal load on each micro-asperity and the area distribution function across the corresponding critical area ranges. Thus, the total normal load on the contact surface is

(20) $$\begin{array}{ll} F_{n} & =F_{ne}+F_{np}=\int_{a_{c}}^{a_{l}}F_{e}(a)n(a)da+\int_{0}^{a_{c}}F_{p}(a)n(a)da\\ & =\frac{4\sqrt{\pi }}{3\left(3-2D\right)}G^{D-1}\lambda D\psi^{\left(2-D\right)/2}E\sqrt{\ln\gamma }{a_{l}}^{D/2}\left({a_{l}}^{\left(3-2D\right)/2}-{a_{ec}}^{\left(3-2D\right)/2}\right)+\lambda H\frac{D}{2-D}\psi^{\left(2-D\right)/2}{a_{l}}^{D/2}{a_{c}}^{\left(2-D\right)/2} \end{array}$$

Stiffness represents a body’s resistance to elastic deformation. As a result, the tangential stiffness of a contact pair is mainly determined by micro-asperities undergoing elastic and elastic–plastic deformation. The tangential stiffness $K_{t}$ of a single elastically deforming micro-asperity is given as follows [41]:

(21) $$K_{t}=\frac{8G^{\prime }a^{1/2}}{\pi^{1/2}}\cdot {\left(1-\frac{Q_{x}}{\mu P}\right)}^{\frac{1}{3}}$$

The tangential load $Q_{x}$, normal load P, and micro-contact area a of a single asperity are approximately related to the total tangential load T, total normal load $F_{n}$, and the real contact area $A_{r}$ of the interface [38]:

(22) $$\frac{P}{a}=\frac{F_{n}}{A_{r}},\frac{Q_{x}}{a}=\frac{T}{A_{r}}$$

Therefore, the proportionality $Q_{x}/(\mu P)$ = $T/(\mu F_{n})$ holds, where *μ* is the friction coefficient.

Thus, the tangential stiffness *k_τe_* for an elastically deforming micro-asperity, considering the influence of the overall tangential load *T* can be expressed as follows:

(23) $$k_{\tau e}=\frac{8G^{\prime }a_{e}^{1/2}}{\pi^{1/2}}(1-\frac{T}{\mu F_{n}}{)}^{1/3}$$

where $G^{\prime }$ is the equivalent shear modulus, given by $G^{\prime }=[(2-\nu_{1})/G_{1}+(2-\nu_{2})/G_{2}{]}^{-1}$, with $G_{1}=E_{1}/[2(1+\nu_{1})]$.

By integrating the stiffness contributions from all elastically deforming asperities, the total tangential stiffness $K_{\tau }$ of the contact pair is obtained as follows:

(24) $$K_{\tau }=\int_{a_{c}}^{a_{l}}k_{\tau e}n(a)da=\frac{8G^{\prime }}{\pi^{1/2}}(1-\frac{T}{\mu F_{n}}{)}^{1/3}\lambda \frac{D}{1-D}\psi^{(2-D)/2}a_{l}^{D/2}\left({a_{l}}^{(1-D)/2}-{a_{c}}^{(1-D)/2}\right)$$

where $k_{\tau e}$ represents the tangential stiffness of a single elastically deforming micro-asperity, and $K_{t}$ is the total tangential stiffness of the contact pair.

## 4. Modeling the Torsional Stiffness of the Joint Traction System

**Figure 3:**
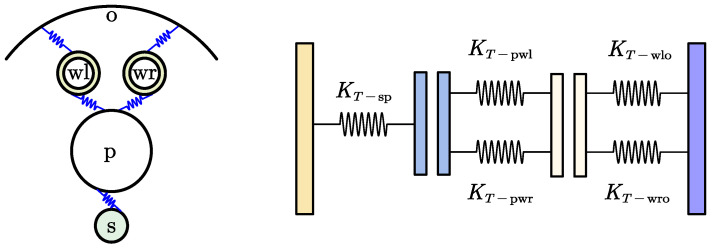
Schematic of torsional stiffness in a single transmission chain.

When the sun roller functions as the input and the planet carrier as the output, the overall torsional stiffness of the joint traction system is modelled as the combined stiffness of three primary subsystems connected in series along a single transmission path: the sun roller–planet roller interface, the planet roller–loading roller interface, and the loading roller–outer ring interface. Figure 3 illustrates a schematic of the torsional stiffness components along this transmission path.

The overall torsional stiffness, $K_{T}$, of the joint traction system, accounting for the contributions from the three parallel transmission paths, can be determined from the torsional stiffness of the individual subsystems referenced to the input (sun roller) as [42]

(25) $$K_{T}=3\times \frac{1}{\frac{1}{K_{T-sp}}+\frac{1}{K_{T-pw}}+\frac{1}{K_{T-wo}}}$$

where $K_{T-sp}$ represents the torsional stiffness of the sun–planet roller subsystem, equivalent to the sun roller; $K_{T-pw}$ represents the torsional stiffness of the planet roller–loading roller subsystem, equivalent to the sun roller; and $K_{T-wo}$ represents the torsional stiffness of the loading roller–outer ring subsystem, equivalent to the sun roller.

These equivalent stiffnesses are derived from the local torsional stiffnesses of the respective subsystems, taking into account the transmission ratios (squared radius ratios). Let $K_{T’-pw}$ be the combined local torsional stiffness at the planet roller due to interactions with both loading rollers and $K_{T’-wo}$ be the combined local torsional stiffness at the loading rollers due to interactions with the outer ring (see conversion formula below). The stiffnesses are then transformed to the sun roller reference frame using the appropriate transmission ratio $K_{T-sp}$ is typically calculated from the tangential stiffness $K_{t-sp}$ using the sun roller radius $R_{s}$ (see conversion Equation (30)).

(26) $$K_{T-pw}=K_{T^{\prime }-pw}{\left(\frac{R_{s}}{R_{p}}\right)}^{2}$$

(27) $$K_{T-wo}=K_{T^{\prime }-wo}{\left(\frac{R_{s}}{R_{w}}\right)}^{2}$$

(Note: In the equations above, $K_{T’-pw}$ and $K_{T’-wo}$ represent the combined local torsional stiffnesses before being referenced to the sun roller, as defined by the sums below. $R_{s}$, $R_{p}$, and $R_{w}$ are the radii of the sun roller, planet roller, and loading roller, respectively.)

The combined local torsional stiffness $K_{T’-pw}$ results from the parallel interaction between the planet roller and the left and right loading rollers:

(28) $$K_{T^{\prime }-pw}=K_{T-pwr}+K_{T-pwl}$$

Similarly, the combined local torsional stiffness $K_{T’-wo}$ results from the parallel interaction between the left and right loading rollers and the outer ring system, as follows:

(29) $$K_{T^{\prime }-wo}=K_{T-wro}+K_{T-wlo}$$

where $K_{T-pwl}$ and $K_{T-pwr}$ are the local torsional stiffnesses of the systems involving the planet roller with the left and right loading rollers, respectively; $K_{T-wlo}$ and $K_{T-wro}$ are the local torsional stiffnesses of the systems involving the left and right loading rollers with the outer ring, respectively. These individual torsional stiffnesses ($K_{T-pwl}$, $K_{T-pwr}$, $K_{T-wlo}$, $K_{T-wro}$) are calculated from their corresponding tangential stiffnesses ($K_{t-pwl}$, $K_{t-pwr}$, $K_{t-wlo}$, $K_{t-wro}$) using the appropriate roller radius.

The general formula for converting tangential stiffness ($K_{t}$) to torsional stiffness ($K_{T}$) for a specific contact pair is as follows:

(30) $$K_{T}=K_{t}R^{2}$$

where *R* is the radius of the roller associated with the stiffness being converted.

## 5. Discussion

### 5.1. Analysis of Factors Affecting the Tangential Stiffness of a Single Roller Contact Pair

**Figure 4:**
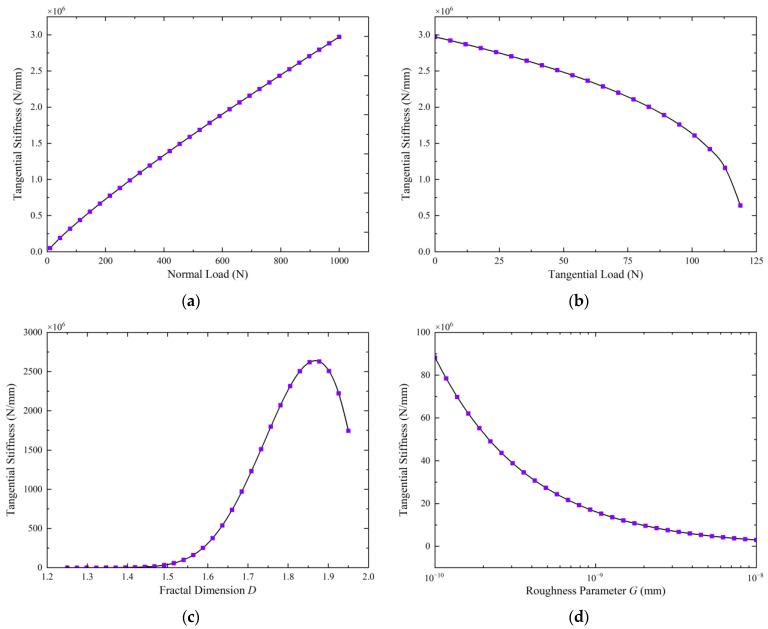

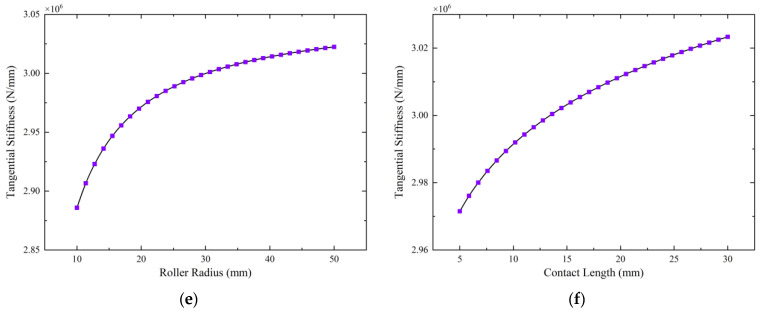
Analysis of factors influencing the tangential stiffness of a contact pair (**a**) Normal load vs. tangential stiffness; (**b**) Tangential load vs. tangential stiffness; (**c**) Fractal dimension vs. tangential stiffness; (**d**) Roughness parameter vs. tangential stiffness; (**e**) Contact length vs. tangential stiffness; (**f**) Roller radius vs. tangential stiffness.

To gain a comprehensive understanding of the torsional stiffness characteristics of the joint traction system, this section first analyses the factors influencing the tangential stiffness of a single roller contact pair. With other parameters held constant (specific values are provided in Table 1), the simulation parameters are selected to represent realistic rehabilitation robot operating conditions. Fractal dimension *D* = 1.4 (range 1.3–1.95) represents typical machined surface characteristics [22,25], while fractal roughness *G* = 1×10^−10^–1×10^−8^ mm corresponds to precision-manufactured metallic surfaces [26,30]). As illustrated in Figure 4a, the tangential stiffness of the contact pair increases non-linearly with the increase in normal load (*F_n_*). This trend occurs because a higher normal load promotes contact between more micro-asperities, thereby increasing the real contact area and enhancing the resistance to tangential deformation.

As illustrated in Figure 4b, the tangential stiffness of the contact pair decreases non-linearly as the tangential load (*T*) increases. When the tangential load exceeds a critical value ($\mu F_{n}$, where μ is the friction coefficient, and $F_{n}$ is the normal load), the tangential stiffness rapidly drops to zero, signaling the onset of macroscopic sliding at the contact interface.

Figure 4c illustrates the influence of the fractal dimension D on tangential stiffness. The results show that for smaller values of D, the tangential stiffness increases significantly as D increases. This trend occurs because under a given preload or normal load, initial contact mainly occurs at the peaks of a limited number of larger micro-asperities, resulting in a relatively small real contact area. As *D* increases, the surface complexity increases, featuring additional scales and denser micro-asperities—particularly a significant rise in the number of medium- and small-sized asperities. This enhanced surface texture allows more micro-asperities to contribute to the contact under the same normal load, thereby increasing the total real contact area. Consequently, the surface generally exhibits a greater capacity to sustain shear forces, resulting in higher tangential stiffness. However, when *D* exceeds approximately 1.85, the tangential stiffness begins to decrease, probably because the surface becomes excessively “sharp” as *D* approaches its theoretical upper limit. Such surfaces are dominated by numerous extremely small, potentially pointed micro-asperities, leading to greater stress concentrations that promote local plastic deformation, ultimately reducing the overall stiffness of the fractal surface.

Figure 4d illustrates that the tangential stiffness gradually decreases as the roughness parameter *G* increases. A larger roughness parameter indicates a rougher contact surface. For a given load, a rougher surface leads to a smaller real contact area, reducing the number of micro-asperities involved in resisting shear and thereby reducing the contact interface’s ability to resist deformation.

Figure 4e,f illustrates the impact of geometric dimensions (contact length *L* and roller radius *R*) on the tangential stiffness of the contact pair. The analysis shows that increases in both contact length and roller radius enhance the tangential stiffness. This variation is also non-linear, though the extent of the influence is relatively limited compared with the influence of other parameters.

### 5.2. Analysis of Torsional Stiffness in the Rehabilitation Robot Joint Traction Drive System

**Figure 5:**
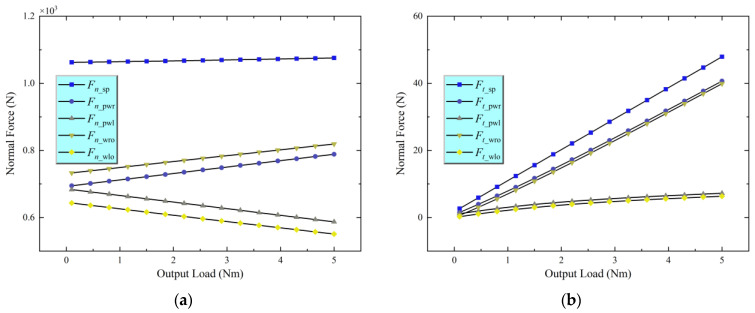
Load in the contact zone. (**a**) Normal load; (**b**) Tangential load.

Before analyzing the overall torsional stiffness, the study first solves the system’s mechanical model. With the sun roller rotating clockwise as the input and under operating conditions of an input speed of 6000 rpm and an output load of 0~5 Nm, the normal ($F_{n}$) and tangential ($F_{t}$) loads within the various contact zones of the joint traction system are determined based on the mechanical model developed in Section 2. The results are illustrated in Figure 5. In the figure, the subscripts s, p, wr, wl, and o refer to the sun roller, planet roller, right loading roller, left loading roller, and outer ring, respectively. For example, $F_{n-sp}$ represents the normal load between the sun roller and the planet roller, and a similar notation applies to the other variables. Key geometric parameters of the transmission components are provided in Table 2.

The analysis of the normal forces in Figure 5a shows that as the output load increases, the normal forces at the sun–planet roller, planet–right loading roller, and right loading roller–outer ring contacts continuously rise. In contrast, the normal forces at the planet–left loading roller and left loading roller–outer ring contacts gradually decrease. This behavior can be explained as follows: as the output load increases, the right loading roller, influenced by frictional forces, tends to shift toward the converging side of the wedge space, leading to an increase in its normal force. Conversely, the left loading roller moves toward the diverging side of the wedge space, causing its normal forces to decrease. The tangential forces analysis depicted in Figure 5b illustrates that the tangential forces in all contact zones increase with the output load, with more significant changes observed in the sun–planet roller, planet–right loading roller, and right loading roller–outer ring contacts.

**Figure 6:**
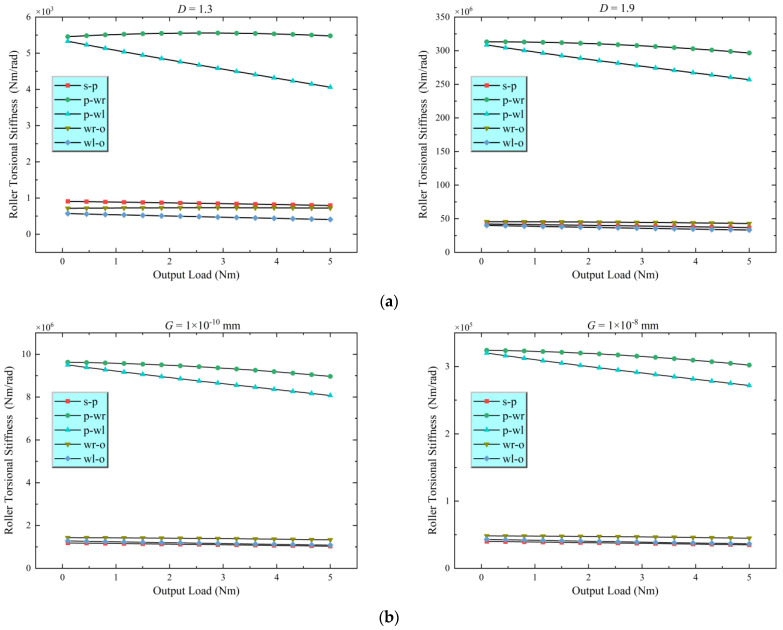
Torsional stiffness of contact pair rollers. (**a**) Effect of fractal dimension D on torsional stiffness for different contact pairs; (**b**) Effect of roughness parameter *G* on torsional stiffness for different contact pairs.

According to the calculated normal and tangential forces, the torsional stiffness of the driving element in each contact pair is computed using Equations (24) [from Section 3, tangential stiffness $K_{t}$] and (30) [conversion to $K_{T}$]. Figure 6 illustrates the influence of the fractal dimension D and roughness parameter G on the torsional stiffness of the five primary contact pairs. The results indicate that the roller torsional stiffness tends to increase as D increases and G decreases. For the same fractal parameters, the torsional stiffness values for the planet–right loading roller and planet–left loading roller contacts are nearly the same. Similarly, the torsional stiffness for the right loading roller–outer ring and left loading roller–outer ring contacts show comparable characteristics. This similarity can be attributed to the nearly identical geometric dimensions of these contact pairs and the relatively similar load levels they experience under the studied operating conditions (Figure 5). Furthermore, the torsional stiffness of the sun–planet roller contact is significantly higher than that of the other contact pairs, mainly because the radius of the planet roller is larger than those of the other rollers; moreover, as per Equation (30), the conversion from tangential stiffness to torsional stiffness involves multiplying by the square of the radius, which significantly amplifies the torsional stiffness associated with the planet roller.

**Figure 7:**
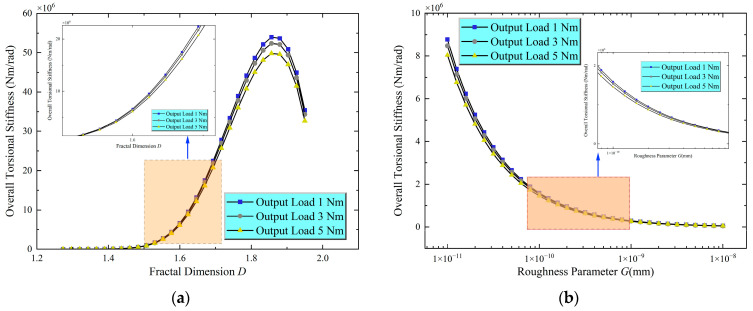
Overall torsional stiffness of the machine. (**a**) Fractal dimension vs. torsional stiffness; (**b**) Roughness parameter vs. torsional stiffness.

After the torsional stiffness for each contact pair’s roller is obtained, the overall torsional stiffness of the machine is calculated by combining these values using Equations (25)–(30). Figure 7a illustrates the trend of the overall joint torsional stiffness as a function of the fractal dimension D under different output loads. Consistent with the behaviour of the tangential stiffness of the contact pairs, the overall torsional stiffness follows a non-monotonic relationship with D: the tangential stiffness initially increases significantly and then decreases after D reaches approximately 1.85. This behaviour aligns with the previous analysis of contact pair tangential stiffness: moderate D values enhance the real contact area, thereby increasing torsional stiffness, while excessively high D values result in surface “sharpness,” leading to stress concentrations and local plastic deformation, ultimately reducing stiffness.

Figure 7b illustrates the effect of the roughness parameter G on the overall torsional stiffness. The results show that as G increases, the overall stiffness gradually decreases. This behaviour is consistent with the impact of G on contact pair tangential stiffness: rougher surfaces result in smaller real contact areas and reduced resistance to deformation.

Furthermore, Figure 7a,b indicates that as the output load increases, the overall torsional stiffness experiences a slight decrease, although the numerical changes are relatively small. This trend can be attributed to the significant initial system preload. As the output load increases, the increase in normal forces across the contact zones is minimal compared with the initial preload, contributing only slightly to stiffness enhancement. However, the increase in tangential forces is more significant relative to the initial tangential forces, leading to a more noticeable reduction in stiffness (Figure 5). The combined effect of these changes in normal and tangential forces results in a slight decrease in overall torsional stiffness as the output load increases.

**Figure 8:**
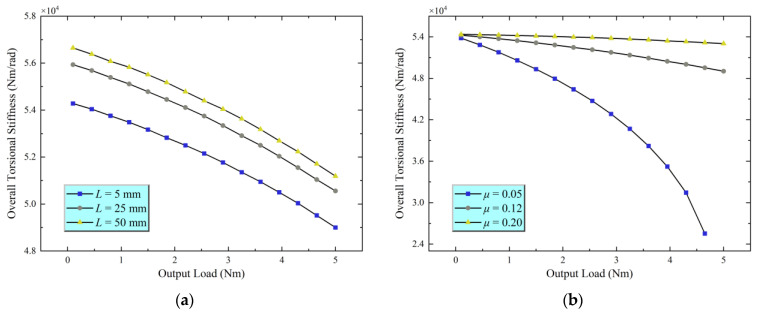
Analysis of the influence of contact length and radius. (**a**) Contact length vs. torsional stiffness; (**b**) Friction coefficient vs. torsional stiffness.

Additionally, this study examines the impact of contact length and friction coefficient on the overall system stiffness. As illustrated in Figure 8a, the overall torsional stiffness continues to exhibit a slight decrease with increasing output load, as previously discussed. However, under the same output load conditions, increasing the effective contact length significantly enhances the overall torsional stiffness. This improvement is mainly due to the longer contact line, which allows for better stress distribution across the contact zone, increases the real contact area, and enables more efficient transmission of tangential forces, ultimately improving the system’s resistance to torsional deformation.

As illustrated in Figure 8b, with other parameters kept constant, the effect of the friction coefficient on overall torsional stiffness demonstrates non-monotonic behavior. First, as the friction coefficient increases, the torsional stiffness increases slowly. However, once the friction coefficient reaches a certain threshold, its effect on increasing torsional stiffness reduces and begins to saturate. This phenomenon can be explained as follows: at lower friction coefficients, relative sliding at the interface occurs more easily, which limits improvements in stiffness. As the friction coefficient increases, more micro-asperity contact points transition from a sliding state to a sticking (adhesive) state, enhancing shear force transmission across the interface and thereby increasing torsional stiffness. However, once most of the potential contact points have shifted to the sticking state, further increases in the friction coefficient no longer significantly enhance the effective sticking contact area. Consequently, the enhancement of torsional stiffness weakens and eventually reaches a saturation point.

The fractal-based torsional stiffness model provides actionable insights for rehabilitation robot joint design and manufacturing. Based on our parametric analysis, optimal torsional stiffness can be achieved by maintaining fractal dimension D between 1.7 and 1.85 and minimizing roughness parameter G, which requires careful control of surface finishing processes to achieve the desired fractal characteristics. Design engineers should consider the trade-offs between contact length L and system compactness, as while longer contact lengths enhance stiffness, they must be balanced against weight and volume constraints critical for rehabilitation robot applications. Similarly, roller radius R shows a positive correlation with stiffness but must be optimized within space limitations. Adequate initial preload (proportional to maximum expected load) should be maintained to ensure consistent performance under varying output loads. From a manufacturing perspective, achieving target fractal parameters requires controlled processes such as precision grinding with appropriate wheel selection and surface treatments for fine-tuning surface characteristics. Quality control should incorporate fractal dimension measurement using surface profilometry to ensure consistency with design specifications, while material selection must balance mechanical properties (E, H) with machinability requirements. For practical implementation, we recommend a systematic approach combining model-based design optimization with prototype testing and iterative refinement to validate performance under actual operating conditions, thus bridging the gap between theoretical predictions and industrial application.

## 6. Conclusions

This study presents a mechanical model for rehabilitation robot joint traction systems and applies fractal theory to develop a contact pair tangential stiffness model that accounts for both elastic and plastic deformation of transmission components. This model is employed to calculate and analyze the influences of normal and tangential loads, fractal parameters (fractal dimension D and roughness parameter G), and geometric dimensions (contact length L and roller radius R) on the torsional stiffness of the contact pair rollers. Finally, a prediction method for the overall system torsional stiffness is proposed according to the results of the contact pair torsional stiffness model. The effects of fractal parameters, output load, component dimensions, and friction coefficient on the overall system torsional stiffness are further examined. The main conclusions are as follows:

1. Contact Pair Torsional Stiffness Characteristics: The tangential stiffness of the contact pair increases non-linearly with the normal load and decreases non-linearly with the tangential load. Once the tangential load exceeds a critical value, the stiffness rapidly drops to zero, and macroscopic sliding occurs. The influence of the fractal dimension D on tangential stiffness follows a non-monotonic pattern, initially increasing and then decreasing, with a peak observed around D ≈ 1.85. An increase in the roughness parameter G leads to a gradual reduction in tangential stiffness. An increase in both contact length L and roller radius R non-linearly enhances tangential stiffness, although their relative influence is limited;
2. Relationship between Overall Torsional Stiffness and Fractal Geometric Parameters: The overall system torsional stiffness also exhibits a non-monotonic dependence on the fractal dimension D, showing an initial increase followed by a decrease, consistent with the trend observed for contact pair tangential stiffness. An increase in the roughness parameter G leads to a gradual reduction in overall system torsional stiffness. Increases in the effective contact length and roller radius significantly enhance the overall system torsional stiffness;
3. Effect of Output Load on Overall Torsional Stiffness: Under conditions of a sufficiently high initial preload, the overall system torsional stiffness exhibits a slight decrease as the output load increases. This trend is mainly due to the combined effect of a relatively small increase in normal load compared with the initial preload and a relatively large increase in tangential load relative to the initial tangential load;
4. Effect of Friction Coefficient on Overall Torsional Stiffness: The influence of the friction coefficient (μ) on the overall system torsional stiffness is non-monotonic. As the friction coefficient increases, the torsional stiffness initially increases gradually. However, once the friction coefficient reaches a certain threshold, its effect on enhancing torsional stiffness reduces and tends toward saturation. This behavior is related to the transition between sliding and sticking states at the contact interface.

The rehabilitation robot joint torsional stiffness model developed in this study, based on fractal theory, provides a more comprehensive consideration of surface roughness effects, offering a theoretical foundation for more precise joint stiffness predictions. The results from the parametric analysis reveal the influence patterns of key design parameters on joint torsional stiffness, providing valuable theoretical guidance and an engineering basis for the design optimization, performance prediction, and control strategy development of high-performance rehabilitation robots.

While the proposed model provides valuable insights into torsional stiffness prediction for rehabilitation robot joints, it is important to acknowledge its limitations, which also pave the way for future research.

First, the model assumes statistically isotropic surfaces and idealized hemispherical asperities, which may not fully represent real machined surfaces with anisotropic roughness patterns and complex asperity geometries. Future work should extend the model to account for these realistic surface characteristics.

Second, our analysis does not consider lubrication or progressive wear effects present during long-term operation. Integrating these tribological factors would enhance the model’s comprehensiveness.

Finally, experimental validation is essential to verify the model’s quantitative predictions against actual rehabilitation robot joint measurements, which we plan to pursue in future research.

## Figures and Tables

**Table 1:** Simulation parameters.

Cases	*F_n_* (N)	*T* (N)	*D*	*G* (mm)	*R*_1_*/R*_2_ (mm)	*L* (mm)	*σ* (MPa)
case a	0~1000	0	1.4	1×10^−8^	10/20	5	450
case b	1000	0~119	1.4	1×10^−8^	10/20	5	450
case c	1000	0	1.3~1.95	1×10^−8^	10/20	5	450
case d	1000	0	1.4	1×10^−10^~1×10^−8^	10/20	5	450
case e	1000	0	1.4	1×10^−8^	10/10~10/50	5	450
case f	1000	0	1.4	1×10^−8^	10/20	5~30	450

**Table 2:** Transmission component parameters.

Parameter	Sun Roller—Planet Roller	Planet Roller—Load Roller	Load Roller—Outer Ring
yield strength (MPa)	450	450	450
contact Radii (mm)	4/13.5	13.5/5	5/40
contact line length (mm)	5	5	5

## Data Availability

The original contributions presented in this study are included in the article. Further inquiries can be directed to the corresponding author.

## Abbreviations

The following abbreviations are used in this manuscript:

PTD	Planetary traction drive
